# HIV-1 Transmission during Early Antiretroviral Therapy: Evaluation of Two HIV-1 Transmission Events in the HPTN 052 Prevention Study

**DOI:** 10.1371/journal.pone.0071557

**Published:** 2013-09-24

**Authors:** Li-Hua Ping, Cassandra B. Jabara, Allen G. Rodrigo, Sarah E. Hudelson, Estelle Piwowar-Manning, Lei Wang, Susan H. Eshleman, Myron S. Cohen, Ronald Swanstrom

**Affiliations:** 1 Lineberger Comprehensive Cancer Center, University of North Carolina at Chapel Hill, Chapel Hill, North Carolina, United States of America; 2 Department of Biology, University of North Carolina at Chapel Hill, Chapel Hill, North Carolina, United States of America; 3 National Evolutionary Synthesis Center, Durham, North Carolina, United States of America; 4 Department of Pathology, Johns Hopkins University School of Medicine, Baltimore, Maryland, United States of America; 5 Vaccine and Infectious Disease Division, Fred Hutchinson Cancer Research Center, Seattle, Washington, United States of America; 6 Division of Infectious Diseases, University of North Carolina at Chapel Hill, Chapel Hill, North Carolina, United States of America; 7 UNC Center For AIDS Research, University of North Carolina at Chapel Hill, Chapel Hill, North Carolina, United States of America; 8 Department of Biochemistry and Biophysics, University of North Carolina at Chapel Hill, Chapel Hill, North Carolina, United States of America; University of Pittsburgh, United States of America

## Abstract

In the HPTN 052 study, transmission between HIV-discordant couples was reduced by 96% when the HIV-infected partner received suppressive antiretroviral therapy (ART). We examined two transmission events where the newly infected partner was diagnosed after the HIV-infected partner (index) initiated therapy. We evaluated the sequence complexity of the viral populations and antibody reactivity in the newly infected partner to estimate the dates of transmission to the newly infected partners. In both cases, transmission most likely occurred significantly before HIV-1 diagnosis of the newly infected partner, and either just before the initiation of therapy or before viral replication was adequately suppressed by therapy of the index. This study further strengthens the conclusion about the efficacy of blocking transmission by treating the infected partner of discordant couples. However, this study does not rule out the potential for HIV-1 transmission to occur shortly after initiation of ART, and this should be recognized when antiretroviral therapy is used for HIV-1 prevention.

## Introduction

Combination antiretroviral therapy (ART) reduces HIV-1 RNA levels in blood and genital secretions [1], [2], [3]. The probability of HIV-1 transmission is related to HIV-1 RNA levels in the plasma [4], [5] and the genital tract [6]. For these reasons, it seemed likely that treatment of an infected person with ART would reduce transmission to a sexual partner. Several observational studies supported this idea [7], [8], [9], [10]. The HPTN 052 study was specifically designed to measure the degree to which suppressive treatment of an HIV-infected person in a discordant relationship could prevent sexual transmission [4]. In HPTN 052, HIV-infected partners (index) with CD4+ T cell counts >350 and <550 cells/ul, were randomized to receive immediate ART (early ART arm), or to receive ART when their CD4+ T cell count dropped to >200 and <250 cells/ul (delayed ART arm). The study was unblinded in April, 2011. At that time, 27 of 28 virologically-linked transmission events [11] were found to have occurred in the delayed ART arm, demonstrating a greater than 96% reduction in HIV-1 transmission over a median of 1.7 years, ascribed to early ART initiation. However, two transmission events raised the possibility that transmission might still occur after ART initiation: one virologically-linked transmission event in the early ART arm (Case A, 052–1168), and one virologically-linked transmission event in the delayed ART arm that occurred after the index initiated ART in response to a falling CD4+ T cell count (Case B, 052–2899) [11]. We evaluated the timing of HIV-1 transmission in these two cases by analyzing the viral sequence complexity and antibody response in the newly infected partners.

## Materials and Methods

### Ethics statement

This research was performed with appropriate IRB approval which was obtained from 36 different reviewing entities, included in Supporting Information S1. Written informed consent was provided, by all participants.

Plasma samples were collected in the HPTN 052 study. Methods used in the trial for HIV-1 diagnosis and HIV-1 viral load testing were described previously [4]. The extent of maturation of the antibody response was determined by examining the pattern of reactivity with viral proteins in ELISA and western blot format assays; antibody reactivity was interpreted using the timing scale described in Fiebig et al. [12].

HIV-1 RNA was extracted from the plasma samples using the QIAamp Viral RNA Mini Kit (QIAGEN), and was reverse transcribed to produce cDNA using Superscript III Reverse Transcriptase (Invitrogen) and an oligo dT primer. Polymerase Chain Reaction (PCR) amplicons were generated from single cDNA templates using an endpoint dilution strategy [13]; primers were tailored for amplification of the *env* gene from subtype C HIV-1, since both transmission events involved subtype C virus [11]. For each sample, approximately 20 full-length *env* amplicons were generated.

Sequences obtained from those amplicons were used to generate phylogenetic trees using the program BEAST (Bayesian Evolutionary Analysis by Sampling Trees) [14]. BEAST uses a Bayesian Markov Chain Monte Carlo approach, implemented using the program BEAST v.1.6.1 [14], which was used to estimate the time to the most recent common ancestor (MRCA) for each sample. For Case A, this analysis was performed using just the major viral population. We used a fixed mutation rate (1.5×10^−5^) which we have found useful in defining time since transmission for a number of recent transmission events (unpublished data). We also used tip dating given the known dates of sampling, which allowed an output in days rather than generations. Accession numbers for the *env* gene sequences used in the BEAST analysis are KC634109-KC634205.

A second approach, the Poisson Fitter [15], was also used to estimate the extent of early evolution in Case A. The Poisson Fitter was implemented at http://www.hiv.lanl.gov/content/sequence/POISSON_FITTER/poisson_fitter.html using the sequences from the major viral population. A mutation rate of 2.16×10^−5^ per site per generation was used in the model.

## Results and Discussion

**Figure 1:**
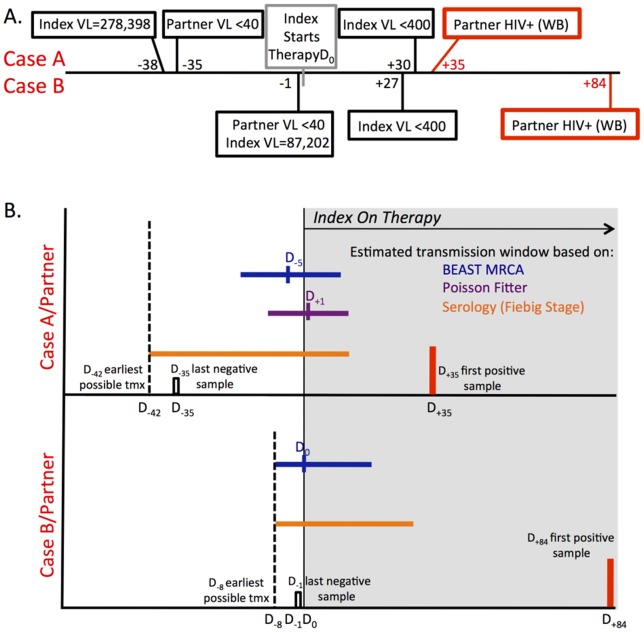
Timeline and summary of data estimating dates of transmission relative to the initiation of therapy. A. A timeline (in days) is shown relative to the date of initiation of therapy (D_0_) for both Case A and Case B. Dates are shown for the proximal time prior to therapy when the viral load in the index was determined along with the proximal date after the initiation of therapy when the index had a viral RNA load below 400 copies/ml. Similarly, the date proximal to but before the date of initiation of therapy when the partner was HIV- (less than 40 copies/ml) and when the partner was diagnosed by western blot analysis (WB) as being HIV+. B. Estimates of the date of transmission to the newly infected partner are shown by the colored horizontal lines on the same timeline as in Panel A, shown separately for Case A and Case B. The gray background indicates the period on therapy for the index. The small black vertical open boxes indicate the dates of the last HIV- sample from each partner, and these boxes are preceded by a black vertical dashed line to indicate the eclipse period (7 days) when transmission could have occurred but not been detected in the blood. The vertical red boxes indicate the dates of the first HIV+ sample in the partners. The horizontal lines represent the range of the possible dates of transmission inferred using the several different approaches. The length of the horizontal line is meant to indicate the uncertainty associated with the estimate. The vertical bar on the horizontal line represents the most likely date of transmission given the sequence diversity. Estimates for the transmission date are shown for serology (in orange), sequence diversity using the Poisson Fitter (in purple), and for expansion of the sequence population in the partner using BEAST (i.e. time to most recent common ancestor/MRCA; in dark blue). For the serology estimate we used the shortest period of time needed for a newly infected person to progress to Fiebig Stage V (23 days) or Fiebig Stage VI) (55 days). Since an infected person can stay in either of these stages for an extended period of time these lines are left-censored at the earliest possible transmission date (dashed black vertical line) given the negative HIV-1 viral RNA load assay plus the eclipse period.

First, we evaluated results obtained from HIV-1 RNA load testing and Western blot testing for anti-HIV antibodies (note that ART initiation is indicated as Day 0; negative numbers indicate days before ART initiation and positive numbers indicate days after ART initiation). In Case A, the partner had undetectable HIV-1 RNA load (<40 copies/ml) at Day −35 and a positive Western blot on Day +35 (Figure 1A). In Case B, the partner had undetectable HIV-1 RNA load on Day −1 and a positive Western blot on Day +84 (Figure 1A). Assuming a 7-day eclipse period between HIV-1 infection and the detection of HIV-1 RNA in plasma, transmission in Case A must have occurred after Day −42 (i.e., an outer limit of 7 days before the last negative sample), and transmission in Case B must have occurred after Day −8. Virologic suppression was first documented in the index at Day +30 and Day +27 for Case A and B, respectively (Figure 1A). This simple analysis indicates that transmission in both cases could have occurred before ART was initiated, or after ART initiation but before virologic suppression occurred.

The dates of transmission were defined further by analyzing viral populations and the antibody response to infection in samples from the newly infected partners to estimate the length of time the newly infected partners had been infected. Viral populations were analyzed using plasma samples collected from the index prior to ART initiation (Case A: Day −38; Case B: Day −1) and plasma samples collected from the newly infected partners on the day of HIV-1 diagnosis (Case A: Day +35; Case B: Day +84).

### Evaluation of transmission in Case A

**Figure 2:**
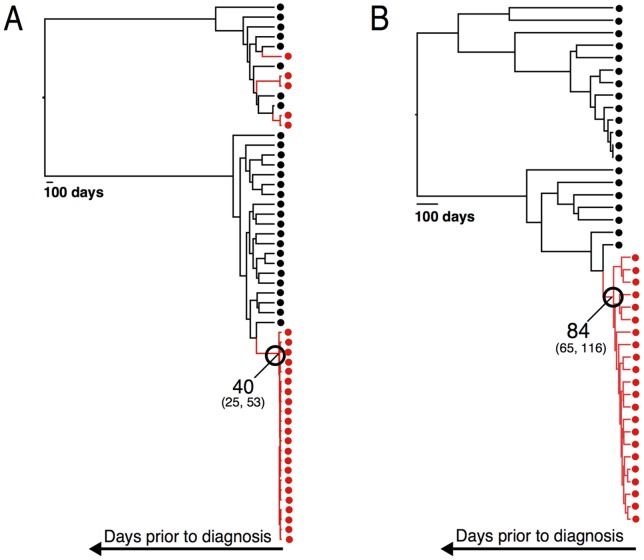
Phylogenetic analysis of viral populations in the index and partner. The program BEAST was used to recreate phylogeny (Bayesian skyline) of the viral *env* sequences generated from the index (black) and partner (red) for Case A (Panel A) and Case B (Panel B). Each tip (with dot) of the tree is an individual sequence. For Case A, sequences from the partner are dispersed among the sequences from the index, indicating the transmission of at least four variants (seen as four discrete positions in the tree where one or more red dots occur). For Case B, the sequences from the partner represent a single lineage/cluster, indicative of transmission of a single variant. The tree structure moves back in time from right to left as indicated by the arrow at the bottom and with the scale shown for 100 days. The tips of the tree are displaced to correspond to the date the sample was taken that gave rise to those sequences. Each internal node/branch point in the tree represents the inferred most recent common ancestor (MRCA) of all sequences that appear to the right of the node, with the position of each node given an estimated date from the date the partner's infection was detected. The dates (in days) of the MRCA within each partner (interpreted as the date expansion of the virus population started after the transmission event) are indicated (40 days for Case A; 84 days for Case B), and the relevant nodes circled. The numbers in small font indicate the estimated error in the dating of each node. The time to MRCA was estimated from two independent runs which were combined, the HKY substitution model, estimated base frequencies, Gamma site heterogeneity model with 4 gamma categories, a strict clock with a fixed mutation rate of 1.5×10^−5^, partner tip dating, and the Bayesian skyline tree model.

In Case A, the partner was diagnosed with HIV-1 infection 35 days after the index initiated ART (Day +35). Phylogenetic analysis of viral sequences from the index at Day −38 was consistent with a chronic HIV-1 infection (i.e., a complex pattern of HIV-1 variants with significant sequence diversity); in contrast, phylogenetic analysis of viral sequences from the newly-infected partner revealed one major population that was genetically homogeneous and three minor viral populations indicating the transmission of at least four variants (Figure 2A). The limited genetic diversity in the major viral population from the newly infected partner suggested that HIV-1 infection occurred close to the time of HIV-1 diagnosis.

We used BEAST analysis [14] of the major viral population in the newly-partner to estimate the number of days between HIV-1 infection (i.e. the number of days since this lineage existed as a single sequence, referred to as the time to the most recent common ancestor, MRCA) and HIV-1 diagnosis. The time to the MRCA was estimated to be 40 days (95% CI: 25, 53 days, Table 1, Figure 2A). This corresponds to an estimated transmission 5 days before the index started ART (Day −2, 95% CI: Day –18, Day +20).

**Table 1:** Data obtained using BEAST analysis, the Poisson Fitter tool, and serological analysis are expressed as the number of days between the estimated date of HIV-1 transmission and HIV-1 diagnosis in the newly-infected partner. These data are also shown in Figure 1B, expressed as days before or after ART initiation in the index.

Subject	HIV-1 Diagnosis^a^	BEAST MRCA in Partner (95% HPD)^b^	Poisson Fitter (95% CI)^c^	Serology (earliest/average)^d^
Case A	Day +35	40 (25, 53)	34 (22, 45)	Fiebig stage V (23/27)
Case B	Day +84	84 (65, 116)	Not Applicable	Fiebig stage VI (55/97)

a HIV-1 diagnosis indicates the number of days between ART initiation in the index and HIV-1 diagnosis in the partner (first HIV-positive sample).

b Estimate of the time between HIV-1 transmission and HIV-1 diagnosis in the newly-infected partner based on BEAST analysis.

c Estimate of the time between HIV-1 transmission and HIV-1 diagnosis in the newly-infected partner based on use of the Poisson Fitter (Case A only).

d The table shows the earliest and average number of days between HIV-1 transmission and the Fiebig stage of each of the newly-infected partners, based on Western blot analysis. The partner in Case A was Fiebig stage V; the partner in Case B was Fiebig Stage VI [12]; a 7-day eclipse period was included in the calculations.

We also used the Poisson Fitter Tool [15] to estimate the time since transmission of the major population and HIV-1 diagnosis. This tool estimates the number of viral generations based on the accumulation of neutral mutations, which are observed before selection on viral sequences by the host immune response [15]. With this tool, we estimated that the partner was infected 34 days prior to HIV-1 diagnosis (95% CI: 22, 45 days, Table 1). This corresponds to transmission one day after the index started ART (Day +1, 95% CI: Day −10, Day +13), which is close to the estimate provided by BEAST analysis.

As an independent measure of the timing of transmission, we examined the HIV-1 antibody response in the newly infected partner. In Case A, the antibody reactivity pattern in the Western blot at the time of HIV-1 diagnosis was Fiebig Stage V [12], displaying broad reactivity to viral proteins but without reactivity to the integrase protein p31. This reactivity pattern appears after Fiebig Stage IV, which lasts on average until 19 days, but can be as short as 15 days after HIV-1 RNA is detectable in the blood, or approximately 26 days, but as early a 22 days, after transmission after including a 7-day eclipse period. Thus, Fiebig stage V can start as early as 23 days after infection and persists on average about 70 days. Based on these serologic findings, the newly infected partner was likely to have been infected at least at least 23 days prior to HIV-1 diagnosis (Table 1); this corresponds to transmission occurring on or before Day +12.

Figure 1B shows a summary of the time estimates associated with this transmission event relative to the time of ART initiation in the index (Day 0). In this case, data obtained from three separate measures (sequence complexity using BEAST, sequence complexity using Poisson Fitter, and serology) indicate that this transmission event occurred significantly before HIV-1 diagnosis, before or shortly after the index initiated ART.

### Evaluation of transmission in Case B

In Case B, the partner was diagnosed with HIV-1 infection 84 days after the index initiated ART (Day +84). Phylogenetic analysis of sequences from the index at Day −1 was consistent with a chronic HIV-1 infection; in contrast, sequences from the newly infected partner represented a single, monophyletic lineage, consistent with transmission of a single HIV-1 variant (Figure 2B). This population had a much higher level of diversity than the major viral population in Case A, suggesting that a longer time had elapsed between HIV-1 transmission and HIV-1 diagnosis in Case B.

We used BEAST analysis to estimate the number of days between HIV-1 infection and HIV-1 diagnosis in the newly infected partner in Case B. The time to the MRCA was estimated to be 84 days (95% CI: 65, 116 days, Table 1, Figure 2B). This corresponds to transmission on the day the index started ART (Day 0, 95% CI: Day −24, Day +19). It is unlikely the transmission took place earlier than Day −8 given the negative HIV-1 RNA test on Day −1, and the BEAST analysis is consistent with transmission occurring prior to Day +19. In Case B, we did not use the Poisson Fitter tool to provide an independent estimate of the timing of transmission. This tool was designed to measure the accumulation of neutral mutations that are observed before the viral population is subjected to strong selective pressure. Given the complexity of the sequences in the newly infected partner in Case B, we chose not to use Poisson Fitter.

We also examined the HIV-1 antibody response in the newly infected partner. Consistent with the greater genetic complexity of the population in this newly infected partner, results from antibody reactivity in Western blot analysis suggested a more mature host immune response. The reactivity pattern for this partner was consistent with Fiebig Stage VI, reflecting the presence of anti-HIV antibodies that included reactivity to the p31 integrase protein. On average, reactivity to p31 does not appear until 90 days after HIV-1 RNA is detectable, but can appear as early as 48 days after HIV-1 RNA detection. Assuming a 7-day eclipse period, the finding of Fiebig stage VI reactivity in this participant indicates that transmission could have occurred as early as 55 days before HIV-1 diagnosis (Table 1), or Day +29. Transmission could have occurred even before this date since Fiebig Stage VI represents the terminal stage in this scale. These serologic findings are consistent with findings based on viral genetic analysis.

Data obtained for Case B are summarized in Figure 1B. In this case, as in Case A, it is most likely that HIV-1 transmission occurred either before ART initiation, or shortly after ART initiation. However, the longer time interval between ART initiation in the index and HIV-1 diagnosis in the partner (84 days, compared to 35 days in Case A) adds an increased level of uncertainty in estimating the timeframe within which transmission occurred.

## Conclusions

The HPTN 052 study provided unequivocal evidence that ART can prevent HIV-1 transmission [4]. This has led to substantial changes in public policy (www.pepfar.gov/documents/organization/177126.pdf) and has inspired several studies to determine the population level effects of ART for prevention. Results from the HPTN 052 study will determine the durability of ART for prevention. In this report, we demonstrate that two HIV-1 transmission events in HPTN 052 that were documented after the index initiated ART most likely occurred either shortly before ART initiation or before ART could sufficiently suppress viral replication in the genital tract. Combination antiretroviral therapy still requires several weeks for suppression of HIV-1 in blood [2]. These results indicate that HIV-infected patients beginning ART should be informed that several weeks of ART may be needed before the risk of HIV-1 transmission is significantly reduced [4]. Documentation that these two transmission events were likely to have occurred before or shortly after ART initiation (prior to virologic suppression) provides further evidence of the efficacy of suppressive ART for prevention of HIV-1 transmission in serodiscordant couples.

## Supporting Information

Supporting information S1 (PDF)
