# Supplementary material for: HIV-1 Transmission during Early Antiretroviral Therapy: Evaluation of Two HIV-1 Transmission Events in the HPTN 052 Prevention Study
Source: PLoS One. 2013 Sep 24;8(9):e71557. doi: 10.1371/journal.pone.0071557 (PMC3782474; doi:10.1371/journal.pone.0071557)
Supplement: Supporting information S1 — (PDF) [file pone.0071557.s001.pdf]

IRBs/ECs and Other Regulatory Bodies by Site

Site	Affiliated IRBs/ECs and Regulatory Bodies
Porto Alegre, Brazil	• UCLA Office for Protection of Research Subjects: Medical Institutional Review Board<br>• Brazil Ministério da Saúde: CONEP: Comissão Nacional de Ética em Pesquisa<br>• Gerencia de Ensino e Pesuisa: Comitê de Ética em Pesquisa do Grupo Hospitalar Conceição-GHC
Rio de Janeiro, Brazil	• UCLA Office for Protection of Research Subjects: Medical Institutional Review Board<br>• Instituto de Pesquisa Clínica Evandro Chagas: Comitê de Ética em Pesquisa<br>• Brazil Ministério da Saúde: CONEP: Comissão Nacional de Ética em Pesquisa<br>• Grupo Hospitalar Conceição-GHC: Comitê de Ética em Pesquisa
Boston, MA, USA	• Fenway Community Health Center: Fenway Community Health Center Institutional Review Board
Chennai, India	• Fenway Community Health Center: Fenway Community Health Center Institutional Review Board<br>• YRG CARE Institutional Review Board<br>• University of California, San Diego: Human Research Protections Program<br>• Health Ministry Screening Committee (India)
Pune, India	• National AIDS Research Institute (ICMR): National AIDS Research Institute (NARI) Ethics Committee<br>• Johns Hopkins University School of Medicine: Johns Hopkins Medicine Institutional Review Board<br>• Health Ministry Screening Committee (India)
Chiang Mai, Thailand	• Johns Hopkins Bloomberg School of Public Health Institutional Review Boards<br>• Human Experimentation Committee, Research Institute for Health Sciences, Chiang Mai University<br>• Research Ethics Committee, Faculty of Medicine, Chiang Mai University<br>• Ethical Review Committee for Research in Human Subjects Ministry of Public Health, Thailand
Kisumu, Kenya	• Kenya Medical Research Institute: KEMRI National Ethical Review Committee<br>• CDC Atlanta: CDC National Center for HIV/AIDS, Viral Hepatitis, STDs and TB Prevention IRB<br>• Kenya National Pharmacy and Poisons Board (PPB)

Site	Affiliated IRBs/ECs and Regulatory Bodies
Harare, Zimbabwe	• University of California at San Francisco: Committee on Human Research, Office of Research Administration<br>• Medical Research Council of Zimbabwe: Medical Research Council of Zimbabwe (MRCZ) Institutional Review Board<br>• Medicines Control Authority of Zimbabwe (MCAZ)<br>• Research Council of Zimbabwe (RCZ)
Blantyre, Malawi	• University of Malawi College of Medicine: College of Medicine Research & Ethics Committee (COMREC)<br>• Johns Hopkins University Bloomberg School of Public Health: Institutional Review Board
	•

Lilongwe, Malawi	• Malawi Ministry of Health & Population: National Health Sciences Research Committee<br>• University of North Carolina School of Medicine: Committee on the Protection of the Rights of Human Subjects
Gaborone, Botswana	• Botswana Ministry of Health: Health Research and Development Committee<br>• Harvard School of Public Health: Human Subjects Committee
Johannesburg, South Africa	• University of Witwatersrand: Human Research Ethics Committee: Medical<br>• Medicines Control Council (South Africa)
Soweto, South Africa	• University of Witwatersrand: Human Research Ethics Committee: Medical<br>• Medicines Control Council (South Africa)
